# Intraventricular Hemorrhage in Term Neonates: Sources, Severity and Outcome

**Published:** 2015

**Authors:** Ladan AFSHARKHAS, Nasrin KHALESSI, Mohammad KARIMI PANAH

**Affiliations:** 1 Department of Pediatric Neurology, Ali-Asghar Children’s Hospital, Iran University of Medical Science, Tehran, Iran; 2 Division of Neonatology, Ali-Asghar Children’s Hospital, Iran University of Medical Science, Tehran, Iran; 3 Pediatrician, Ali-Asghar Children’s Hospital, Iran University of Medical Science, Tehran, Iran

**Keywords:** Intraventricular hemorrhage, Term neonate, Outcome

## Abstract

**Objective:**

Intraventricular hemorrhage (IVH) occurs in preterm infants; however, the occurrence of this event is less frequent in term neonates. The present study evaluated clinical characteristics, pathophysiological features, and early outcome of term neonates with IVH in a referral neonatal center in Iran.

**Materials & Methods:**

This study was performed on 30 full-term neonates admitted to the Neonatal Intensive Care Unit (NICU) of Ali-Asghar Hospital, Tehran, Iran between March 2005 and April 2011. IVH was diagnosed using cranial ultrasonography, or brain magnetic resonance imaging (MRI).

**Results:**

The mean age at onset of symptoms was 3.9 days. Seizure was the commonest clinical symptoms followed by poor feeding and fever. The sources of bleeding in the brain were choroid plexus (60%), germinal matrix (20%) and parenchyma (6.7%). Severity of bleeding included 33.3% grade I, 30.0% grade II, 36.7% grade III to IV. Fifteen (50%) cases had coagulopathy. Twenty-five (83.3%) cases were discharged with a good condition, three (10%) cases were referred to surgical ward and two cases (6.7%) died in NICU.

**Conclusion:**

The main source of IVH in term neonates is choroid plexus; the most common clinical symptoms include seizure and poor feeding, and one-third of IVH events are graded as III to IV. Most affected neonates are discharged from NICU without CNS complication, about 10% need to refer to surgical interventions, and death was occurred in a few of neonates.

## Introduction

Despite considerable prevalence of intraventricular hemorrhage (IVH) in preterm newborns, this phenomenon is uncommonly appeared in term neonates and is accompanied with the different underlying etiologies, locations, clinical manifestations, and outcome (1-3). IVH in neonates is usually defined as the occurrence of bleeding in lateral and third or fourth ventricles characterized by hyper-attenuating fluid typically seen as layering within the ventricles in imaging studies (4, 5). Although this type of hemorrhage is primarily specified to preterm condition that might be detected in half of preterm neonates, but some studies have reported the evidences of IVH in about 3.5% to 5.0% of term neonates (6, 7). This discrepancy can be due to the greater maturity of the brain at term and lower rate of underlying risk factors predisposing to bleeding such as coagulative disorders (8). Although the main source and etiology of IVH in about half of the term neonates remained unknown, but mother risk factors such as preeclampsia, urogenital tract infections, chorioamnionitis and some neonatal risks including asphyxia, traumas, vitamin K deficiency, thrombocytopenia and sinovenous thrombotic events have a major role for appearing this event (9). The source of IVH in term and pre-term infants are also different. Although most IVH events in preterm neonates are originated from the fragility of capillaries in the germinal matrix, IVH mostly emanate from residual germinal matrix tissue, the choroid plexus, and the thalamus, in term neonates (10). IVH in both preterm and term neonates has significant consequences, particularly leading to adverse neurodevelopment and even death particularly occurred in early onset IVH (11). The early onset IVH is usually associated with some underlying factors including a lower gestation and birth weight, steroids therapy, antenatal and postnatal complications as well as the mode of delivery (12). Although optimal management and surgical interventions have led to the appropriate outcome in neonates with IVH, but because of severe neurological defects and hemodynamic instabilities, notable number of affected neonates died from this event. The present study was conducted to address clinical characteristics, pathophysiological features, and early outcome of term neonates with IVH in a referral neonatal center in Iran.

## Materials & Methods

This descriptive cross sectional study was approved by medical Ethics Committee of Iran University of Medical Science. Thirty full-term neonates enrolled in the study admitted to the NICU of Ali-Asghar Hospital in Tehran between March 2005 and April 2011. Those neonates with any congenital anomalies, intrauterine growth retardation, or prematurity were excluded from the study. IVH was diagnosed using different imaging methods including cranial ultrasonography, or brain MRI during first 24 hours of admission. All brain-imaging findings were reviewed and finalized by a pediatric radiologist. Findings of IVH were graded as: grade I (the mildest form of IVH that bleeding was limited to the lining of the ventricles), grade II (the blood does spilled into the ventricles, but there was no enlargement or swelling), grade III (the ventricles had become enlarged and are full of blood), and grade IV (Blood spilled out from the ventricles into the surrounding brain). Baseline information were extracted from the hospital recorded files including sex, gestational age, age of onset of symptoms, clinical manifestations, birth weight, history of birth trauma, perinatal asphyxia (apgar score at first and fifth minutes less than 5), neonatal coagulative disorders such as thrombocytopenia (platelet count less than 150×103/mm3), disseminated intravascular coagulopathy (prothrombin time more than 15 second and partial prothrombin time more than 35 second, D-dimer more than 0.5 mg/ml and thrombocytopenia), sinovenous thrombosis (documented by Brain MRI), serum sodium level for assessment of hypernatremia (Na more than 150 meq/l), bleeding points on imaging documents and outcome (15). Post hemorrhagic hydrocephaly and death during hospitalization was considered as poor outcome and without these problems, as good condition. In all cases, coagulative factors were checked three months after discharge. This period was necessary because patients received fresh frozen plasma (FFP) and other blood products during hospitalization and it might disturb results. For diagnosis of IVH, first, cranial ultrasonography was performed and then serial cranial ultrasonographies were done for more following the progression of IVH. In some cases, brain MRI was ordered for better evaluation of hydrocephaly, sinovenous thrombosis and concomitant brain structural anomalies. Results were presented as mean and standard deviation for quantitative variables and were summarized by absolute frequencies and percentages for categorical variables. Categorical variables were compared using chi-square test or Fisher’s exact test. For the statistical analysis, SPSS version 16.0 (Chicago, IL, USA) was used. P values of 0.05 or less were considered statistically significant.

## Results

The study group comprised of 18 (60.0%) boys and 12 (40.0%) girls, with the mean gestational age of 38.7±0.86 weeks. The mean age at onset of symptoms was 3.9 days with median of 2 days. The average of birth weight was 3211±512.51 grams. Only two neonates had birth weight lower than 2500 grams. There were no cases of asphyxia or birth trauma. Seizure (in 15 cases) was the commonest clinical symptoms followed by poor feeding (in 9 cases) and fever (in 3 cases) and other signs in three patients. The sources of bleeding in the studied neonates were choroid plexus (60.0%), followed by germinal matrix (20.0%) and brain parenchyma (6.7%) (Table 1). About half of the bleedings appeared in the right sides and only in 36.7% was visible in the left side. Regarding grading of bleeding, 33.3% of bleedings was graded as I and 30.0% was graded as II, while grade III to IV was specified to 11 neonates (36.7%). Analysis of differences in the age of onset of symptoms across groups with the different severities of bleeding revealed statistically significant differences so that the symptoms in neonates with higher grades of bleeding was later appeared (P=0.003) as shown in Table 2. In addition, the bleedings with higher grades were originated more from choroid plexus or parenchyma (P=0.001). Besides, the bleedings with lower grades were more specified to germinal matrix. Fifteen (50%) neonates had coagulation disorders including DIC 6 (20%), sinovenous thrombosis 5 (16.7%) and thrombocytopenia 4 (13.3%) of cases. There was dehydration and hypernatremia in three cases with sinovenous thrombosis. Assessment of coagulative factors three months after receiving blood products showed protein C deficiency in one case. Among all study neonates, 25 (83.3%) were discharged from NICU with good condition and three of them (10%) were referred to surgical ward because of hydrocephaly and finally 2 neonates (6.7%) died in NICU.

**Table 1:** The Characteristics of Intraventricular Bleeding in Patients

**Variable**	**Number (percent)**
**Grade of bleeding**	
I	10 (33.3)
II	9 (30.0)
III	5 (16.7)
IV	6 (20.0)
Side of bleeding	
Left	11 (36.7)
Right	15 (50.0)
Unknown	4 (13.3)
Source of bleeding	
*Choroid plexus*	18 (60.0)
*Germinal matrix*	6 (20.0)
*Parenchyma*	2 (6.7)
Unknown	4 (13.3)

**Table 2:** Association Between the Grade of Bleeding And The Age of Onset of Symptoms And Source of Bleeding

**Variable**	n (%)	n (%)	n (%)	n (%)	P-value
IVH grading	I	II	III	IV	
Age of onset of symptoms					
< 7 days	9 (36.0)	9 (36.0)	5 (20.0)	2 (8.0)	NS*
> 7 days	1 (20.0)	0 (0.0)	0 (0.0)	4 (80.0)	0.003
Source of bleeding					
*Choroid plexus*	4 (22.2)	8 (44.4)	5 (27.8)	1 (5.6)	0.001
*Germinal matrix*	5 (83.3)	1 (16.7)	0 (0.0)	0 (0.0)	NS*
*Parenchyma*	0 (0.0)	0 (0.0)	0 (0.0)	2 (100)	0.001

* NS=Not significant

## Discussion

IVH in neonatal period ranges from a silent bleeding to an extended bleeding into the ventricles or parenchyma. This event commonly occur in preterm infants, especially less than 32 weeks of gestation and in infants with very low birth weight; however the occurrence of this phenomenon is expectable less frequently in term neonates (13). In our observation, most IVH events occurred within the first week of age. In both preterm and term neonates, almost 90% of IVHs occurred within the 72 hours after birth that half of them might occur in the first 24 hours. However, its occurring after the first month of birth is very rare (14). In our study, half of patients had coagulative disorders were including DIC, sinovenous thrombosis and thrombocytopenia. In consistent with our study, in another study brain MRI demonstrated thrombosis in the vein of Galen or major brain venous sinuses in 30% of cases with IVH and hemorrhagic infarct with thrombosis in 25% of cases (13). Fifty-two neonates with sinovenous thrombosis were evaluated and common risk factors were complicated delivery, sepsis, pre-eclampsia and asphyxia. Only one case had dehydration (14). However, in our study, most of cases with sinovenous thrombosis had dehydration, which implies more attention to neonatal feeding and hydration during first weeks of life. We showed that the main source of bleeding was choroid plexus in about two-third of neonates, followed by germinal matrix and parenchyma. It seems that the origins of IVH in preterm and term neonates are discrepant so that the primary locus of IVH in preterm cases is subependymal germinal matrix with its fragile capillaries, while in term neonates, most IVHs arise from the posterior tufts at the glomus in the choroid plexus, and less common arise from the small residual germinal matrix tissue near the thalamocaudate groove, the thalamus, and the watershed area of the foramen of Monro near the caudate nucleus (16, 17). In another study, of 20 studied affected neonates, IVH eliminated from the choroid plexus in nine neonates and subependpmal germinal matrix in other 11 neonates (18). Besides, the thalamus was as the main original site for IVH in 63% of the neonates (19). Apparently, the main source of bleeding directly depends on the main etiology as well as cerebral vascular structure. Seizure in our observation was the most frequent clinical symptoms followed by poor feeding. Focal and generalized seizure is appeared in two-third of neonates with IVH, especially in first 48 hours of birth (20). Other manifestations which are less common include flaccidity, loss of pupillary reaction, loss of extraocular movements, respiratory abnormalities, coma, jitteriness, irritability, vomiting, shrill cry, full fontanel, gaze palsy, central facial weakness, opisthotonic posturing, fever or hypothermia, hypo- or hyperglycemia, decreased lower extremity tone, neck flexor hypotonia, head lag, and brisk reflexes (20). Type and severity of IVH-related symptoms is dependent to the size of hemorrhage, location of bleeding, damage to the surrounding tissues, and other underlying predisposing factors of bleeding. However, IVH in approximately 25 to 50% is remained asymptomatic and only can be discovered on imaging procedures (21). Finally, 6.7% of our neonates died in NICU because of life-threatening events of IVH. The most common complications due to IVH are post-hemorrhagic hydrocephalus, ventriculomegaly with atrophy of brain tissue, and periventricular leukomalacia rarely occurred in the affected neonates, but results in catastrophic results (22). The consequences of IVH are dependent to the grade of bleeding. So that, mortality and sequelae due to IVH, ranged from 5% in grade I to 90% in Grade IV, respectively (22). In our study, three cases were referred to neurosurgeon because of post hemorrhagic hydrocephaly for shunt devising and two cases died due to high grade of hemorrhage and severe brain damage during DIC. There were some limitations in our study including a small sample size and lack of available information about late complications. In conclusion, IVH may occur in term and preterm neonates and can lead to abnormalities in brain growth and development. We postulate that the main source of IVH in term neonates is choroid plexus; the most common clinical symptoms include seizure and poor feeding, and about one-third of IVH events are graded as III to IV. With respect to in-NICU outcome, most affected neonates are discharged from NICU with a good condition, about 10% need to refer to surgical interventions, and death was occurred in a few of neonates.
